# Innovation, Inhibition and Flexibility in Rhesus Macaques (*Macaca mulatta*)

**DOI:** 10.1002/ajp.70027

**Published:** 2025-03-30

**Authors:** Josephine Hubbard, Brenda McCowan

**Affiliations:** ^1^ Animal Behavior Graduate Group University of California Davis Davis California USA; ^2^ School of Veterinary Medicine, Department of Population Health & Reproduction University of California Davis Davis California USA

**Keywords:** behavioral plasticity, exploratory diversity, foraging puzzle, multi‐access box, rhesus monkey

## Abstract

Innovation is a key component of behavioral flexibility. When individuals are presented with novel problems, their ability to behave flexibly often relies upon their exploratory tendencies, motivational states and intrinsic traits. Studies of repeated innovation pose additional benefits to understand mechanisms of behavioral flexibility, including measuring persistence, exploration and inhibitory control when learned solutions are blocked. The multi‐access box (MAB) paradigm tests repeated innovation without prior training and minimal habituation. We tested fifty adult captive female rhesus macaques (*Macaca mulatta*) with a MAB to evaluate the role of individual traits on repeated innovation and explore the relationship between inhibitory responses and innovation. We found that exploratory diversity positively predicted repeated innovation, as has been reported for previous studies. We also found that traits such as age and personality influenced innovation, where younger individuals and those that scored high on nervousness had higher innovation scores. However, we did not find any relationship between inhibitory responses and innovation. Our study provides the first assessment of repeated innovation in rhesus macaques using a MAB design and highlights the importance of individual traits for repeated innovation in this species.

## Summary

- This is the first investigation of repeated innovation in rhesus macaques using a multi‐access puzzle box design, which is useful for assessing the influence of persistence and inhibition on repeated innovation.
- Higher exploratory diversity predicts repeated innovation in rhesus macaques, with young individuals showing higher exploratory diversity scores.
- Younger individuals and those with nervous temperament had higher repeated innovation scores indicating the importance of individual traits on cognitive performance.

## Graphical abstract

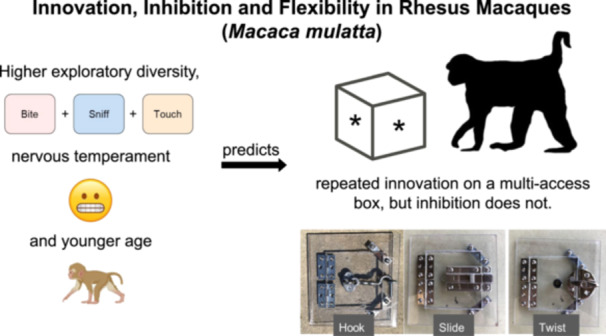

## 1 Introduction

Identifying traits that facilitate innovative and flexible behavior is crucial for understanding how animals cope with environmental change and exhibit resilience to adversity. When conditions are variable, flexibility in behavior can be advantageous due to quick within‐generation responses compared to those shaped by natural selection (Price et al. 2003). Individual variation in cognitive style or temperament can influence key behavioral processes such as foraging and antipredator behavior (Mazza et al. 2019), as well as responses to environmental change (Réale et al. 2007; Cockrem 2022). Understanding both the traits that predict innovation and their underlying processes are valuable for understanding the constraints, costs, and benefits for individuals in a world with changing contingencies (Griffin and Guez 2014; Reader et al. 2016). Additionally, under dynamic conditions individuals are likely to encounter varied innovation opportunities, making sequential or repeated problem‐solving a valuable extension of the study of innovation due to its ecological relevance. Repeated innovation experiments also provide unique opportunities to examine persistence and the ability to inhibit previous responses and express flexibility to explore new solutions. Although studies of repeated innovation in animals have been emerging in recent years on a variety of species, few have explored these processes in monkeys. Here, we measure mechanisms of repeated innovation, inhibition and flexibility using rhesus macaques as a model due to their well‐known resilience and behavioral flexibility. We also explore how individual traits influence the ability to innovate repeatedly using long‐term datasets on rank and temperament.

Macaques are good models of behavioral flexibility because they live across a wide variety of environments (IUCN 2024). Many species of macaques are considered adaptable and have been referred to as “weed species” due to their ability to invade novel urban areas (Richard et al. 1989). The conditions for innovative behavior in primates have been hypothesized to be achieved through exploration, learning and insight which heavily depends on motivational, social and ecological conditions (Kummer and Goodall 1985). Indeed, some macaque species are natural innovators by using novel techniques to process or acquire local foods such as shellfish (Gumert and Malaivijitnond 2012). Additionally, certain species of macaques such as rhesus thrive under captive conditions and are used worldwide for biomedical research (Singh et al. 2021). Despite this potential for using macaques as a model to understand the mechanisms of innovation and flexibility, few studies have been conducted on macaque problem solving outside of biomedical contexts or manipulations. The few studies that have been conducted indicate that problem solving performance in macaques depends on their rearing environment (Capitanio and Mason 2000; Mangalam and Singh 2013), genetic phenotype as a result of selective breeding (Capitanio and Mason 2019), as well as individual traits such as rank, sex, or age (Bunnell and Perkins 1980). For captive rhesus macaques (*Macaca mulatta*), individuals reared with canine companions versus inanimate surrogate mothers showed more consistent problem‐solving techniques on a foraging extraction task, illustrating the importance of rearing environment on cognitive style (Capitanio and Mason 2000). The expression of a persistent behaviorally inhibited phenotype may also be influenced by rearing environment due to the selective breeding of captive animals to achieve certain health statuses such as specific pathogen free (Capitanio and Mason 2019). However, individual traits may also play a role in expressing flexible or temporary inhibitory responses during repeated innovation, as demonstrated by reversal learning performance in long‐tailed macaques (*Macaca fascicularis*) which found that high ranked animals committed more errors after reversal than low ranked animals (Bunnell and Perkins 1980). The only study to date that assesses repeated innovation in macaques is a recent study on Barbary macaques (*Macaca sylvanus*) using a multi‐step foraging problem. However, instead of requiring subjects to inhibit previous responses by locking previously solved solutions, it required them to build upon previously successful responses to maintain success in solving further iterations of the task (Amici et al. 2020). Inhibition in this study was measured simply by attempts to reach directly through the plexiglass box rather than a protocol where individuals must inhibit previously rewarded responses to explore new solutions (Amici et al. 2020). Overall, studies of repeated innovation are lacking across primate species, and vary significantly in the methodologies used.

Although many paradigms have been used to assess problem solving across species, the multi‐access box (MAB) is a promising tool for assessing repeated innovation in free‐ranging and captive animals. This paradigm provides an extractive foraging puzzle with several simultaneous solutions, allowing the subject to explore and solve without any prior knowledge or training. During MAB assessments, key variables that have proven valuable for innovation in previous studies can be measured, including neophobia, persistence and exploratory diversity. Neophobia can influence innovation through an avoidance of novelty, which may inhibit interactions with objects that could lead to innovative responses. Prior studies exploring the role of neophobia on innovation suggest that lower initial neophobia predicts greater success at repeated innovation (Johnson‐Ulrich et al. 2018; Caicoya et al. 1996). In fact, the combination of low neophobia and high rates of innovation can even contribute to the success of invasive species expanding into new habitats (Cohen et al. 2020). Once individuals come into contact with the task, persistence can be measured as the amount of time actively working, and is perhaps the most common factor across species to promote success in MAB contexts (spotted hyenas: Johnson‐Ulrich et al. 2018; Asian elephants: Jacobson et al. 2021; racoons: Daniels et al. 2019; Redfronted lemurs: Huebner and Fichtel 2015; bat‐eared foxes: Petelle et al. 2023; meerkats: Thornton and Samson 2012). However, the quality and diversity of interactions during the task can be measured by exploratory diversity which sums the number of unique behaviors or motor patterns used in the exploration of an object (Daniels et al. 2019; Diquelou et al. 2016; Jacobson et al. 2021; Williams et al. 2021). Additionally, if the MAB is designed such that solutions can be locked then it can provide additional opportunities to test how inhibitory responses influence innovation (Lea et al. 2020). Once learned solutions have been locked, the ability for individuals to inhibit previous responses and explore new options may represent a measure of flexibility in the MAB task. The MAB paradigm has been used to test many captive species (e.g., raccoons: Daniels et al. 2019; Asian elephants: Jacobson et al. 2021; African lions & snow leopards: O'Connor et al. 2022; felids: O'Connor and Vonk 2022; Great apes: Manrique et al. 2013; spotted hyenas: Johnson‐Ulrich et al. 2018; New Caledonia crows & kea: Auersperg et al. 2011; Auersperg et al. 2012), although wild populations have become a focus in recent years (e.g., parrots: Godinho et al. 2020; mouse lemurs: Henke‐von der Malsburg and Fichtel 2018; red‐fronted lemurs: Huebner and Fichtel 2015; spotted hyenas: Johnson‐Ulrich et al. 2020; bat‐eared foxes: Petelle et al. 2023). Although many of these MAB studies do not have large sample sizes, those that do show mixed results regarding the relationship between inhibitory responses and repeated innovation. For example, some studies found no relationship between inhibitory tests such as the detour task and repeated innovation on a MAB (Johnson‐Ulrich et al. 2018; Logan et al. 2022). However, a study on great‐tailed grackles found consistent individual variation between reversal learning measures and repeated innovation on two different MABs (McCune et al. 2023). Additionally, a study on lemurs found that number of errors on a previously locked solution on a MAB predicted repeated innovation (Henke‐von der Malsburg and Fichtel 2018). Interestingly, one study illustrated that some species can outperform others in repeated innovation once solutions are locked, such as kea outperforming New Caledonian crows (Auersperg et al. 2011). Although reversed contingency tasks have been performed indicating that they can inhibit prepotent responses (Murray et al. 2005), these MAB techniques have never been used to test rhesus macaques (*Macaca mulatta*) despite their immense potential as a model of behavioral flexibility.

Using a MAB design, we tested captive adult female rhesus macaques for repeated innovation and inhibitory control as a measure of flexibility. During MAB trials we measured within trial behaviors such as frequency of threats towards the experimenter, presence of stereotypical behavior, as well as exploratory diversity, persistence and number or duration of errors on locked solution types. We included measures such as frequency of threats towards the experimenter and presence of stereotypical behavior because we expected that these behavioral expressions may distract from focusing on solving the MAB, and thus may negatively influence performance. When macaques were first given the MAB, we predicted that individuals with lower neophobia would be more likely to innovate. To solve more than one door on the MAB, individuals must employ flexibility by switching to new solutions and exert inhibitory control by suppressing a previously learned response. Inhibitory control likely enhances success with multiple solutions, where individuals that interact for a shorter duration of time (or lower frequency of interactions) with previously preferred solutions will be more likely to repeatedly innovate. We expected that individuals with higher exploratory diversity, greater persistence and those that committed fewer errors, or spent less time on locked solutions, would show greater success in repeated innovation.

We also explored the relationship between individual traits such as age, health status, rank, and personality traits with repeated innovation. Despite the fact that in other primate species, it was found that adults were more innovative than juveniles (callitrichids: Kendal et al. 2005; lemurs: Huebner and Fichtel 2015), we expected age to have a negative relationship with repeated innovation because our sample is composed solely of adults. Since older adults may experience cognitive decline and/or a decline in motivation to participate in experiments we expected that they would be less likely to repeatedly innovate. Additionally, subjects were split between two health statuses that may be relevant for captive primates. Half the subjects had been bred as a lineage to eliminate specific pathogens (SPF), whereas the other half had not been isolated for specific breeding purposes (conventional). The health status of this population has been suggested to play a role in the expression of behavioral inhibition in captive rhesus macaques (Capitanio and Mason 2019) and was thus tested here for its significance in how individuals approach novel foraging problems. Since rhesus macaques are highly despotic, rank often plays an important role in the acquisition of preferred resources. If priority of access to preferred resources is a major factor that influences solving behaviors, we might expect high ranked individuals to be more likely to innovate. However, if necessity to gain access to resources instead drives solving behavior, we would expect low ranked individuals to be more likely to innovate. Furthermore, subjects that possess certain personality traits may be more likely to innovate as these facets may influence cognitive performance (Carere and Locurto 2011). For example, although personality ratings in chimpanzees did not influence their likelihood to solve foraging puzzles, it did have an influence on how they interacted with novel objects (Hopper et al. 2014). Another study assessing different methodologies for measuring personality found that behavioral observations and experimenter ratings of Asian elephants were more reflective of problem‐solving performance than novel object testing (Barrett and Benson‐Amram 2021). However, the role of personality on innovation in macaques has gone relatively unexplored, so we assessed whether personality assessments during infancy (i.e., behavioral responses to a relocation, responses to novel objects and experimenter ratings) influenced repeated innovation on the MAB as adults. We expected that individuals that are less reactive to the relocation (i.e., lower changes in activity and emotionality across the relocation period), as well as those that interacted more with novel objects or stimuli, will be more successful at repeated innovation. We also expected that individuals that scored high on confidence in experimenter ratings may be more likely to interact with the MAB, whereas those that scored high on gentle may be better able to focus on the task, resulting in greater success in repeated innovation.

## 2 Methods

### 2.1 Study Site and Subjects

All data were collected at the California National Primate Research Center (CNPRC) in Davis, California. The CNPRC is a nonhuman primate research facility associated with the University of California, Davis. Multi‐access puzzle box (MAB) experiments were conducted with 50 adult female rhesus macaques (*Macaca mulatta*). Due to a lack of adult males available for testing, we focused solely on adult females for this study. Females were selected based on outdoor rearing history, despite being indoor pair‐housed at the time of testing. Females were also chosen based on whether they possessed behavioral predictors from previously collected datasets, including rank data and participation in the BioBehavioral Assessment (BBA) program that has been conducted at the CNPRC for over two decades (Golub et al. 2009; Gottlieb et al. 2018; Capitanio et al. 2017). We used these rank and BBA measures as predictors for individuals being tested with the MAB.

**Figure 1:**
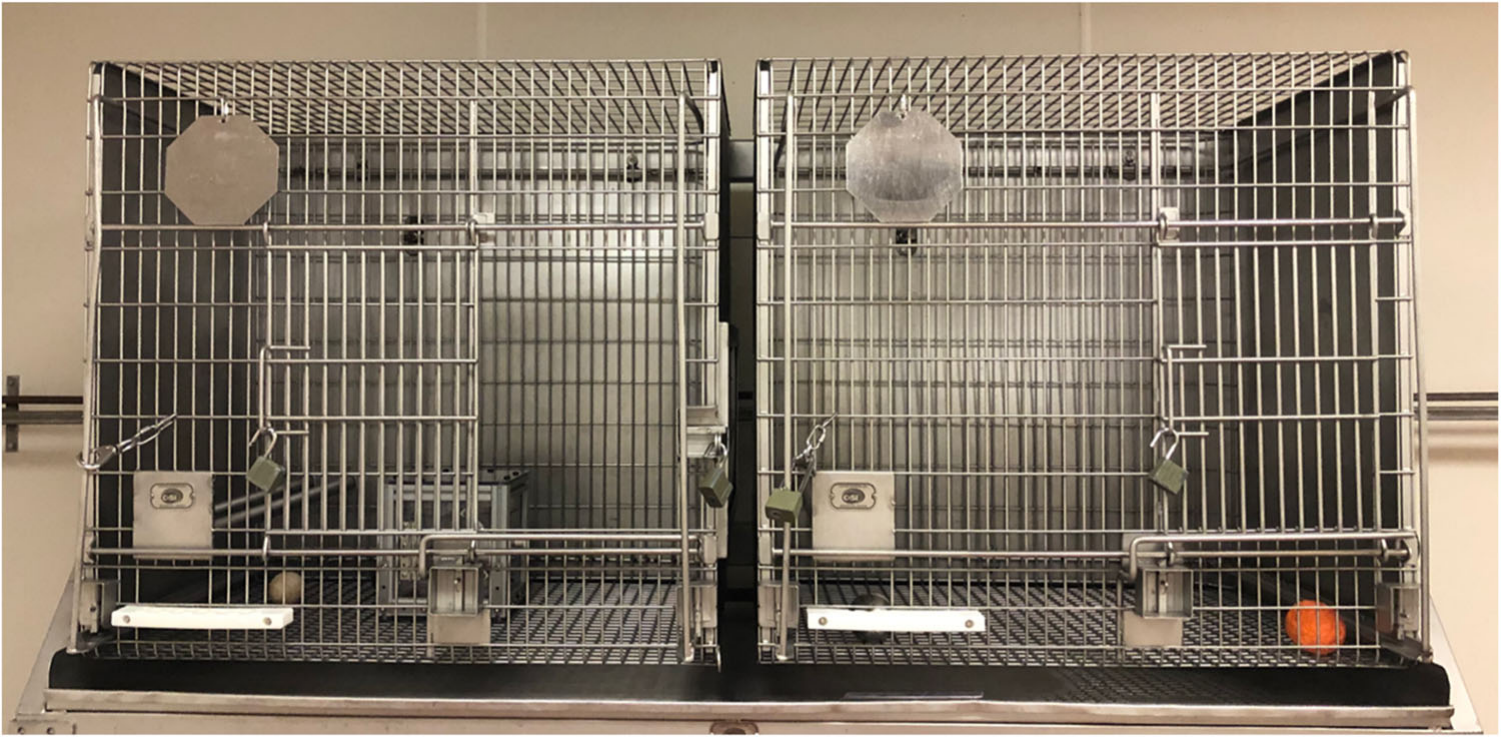
Testing setup with two adjacent enclosures connected by a pairing door, allowing the animal to move between the holding enclosure (right) and the test enclosure with the MAB (left).

Trials were conducted for 2 months, from March 7th to May 19th of 2022. Experimental testing was mainly conducted in the morning, from 8 am to 12 pm, but on a few rare occasions it extended until 2 pm. Subjects were fed twice daily and provided water ad libitum. On the day of testing, subjects were transported from their home cages to the testing cage and given 15 min to acclimate to the new space. The test enclosure was similar to their home cage and was composed of two adjacent compartments separated by a pairing door, allowing to keep test subjects separated from the MAB before the start of the trial (Figure 1). All trial data were video recorded using two cameras, (a SONY Handycam and a Canon Rebel 5) to obtain both zoomed out and close‐up views. Once a subject finished with experimental testing, they were transported back to their home cage.

### 2.2 Multi‐Access Puzzle Box Task

**Figure 2:**
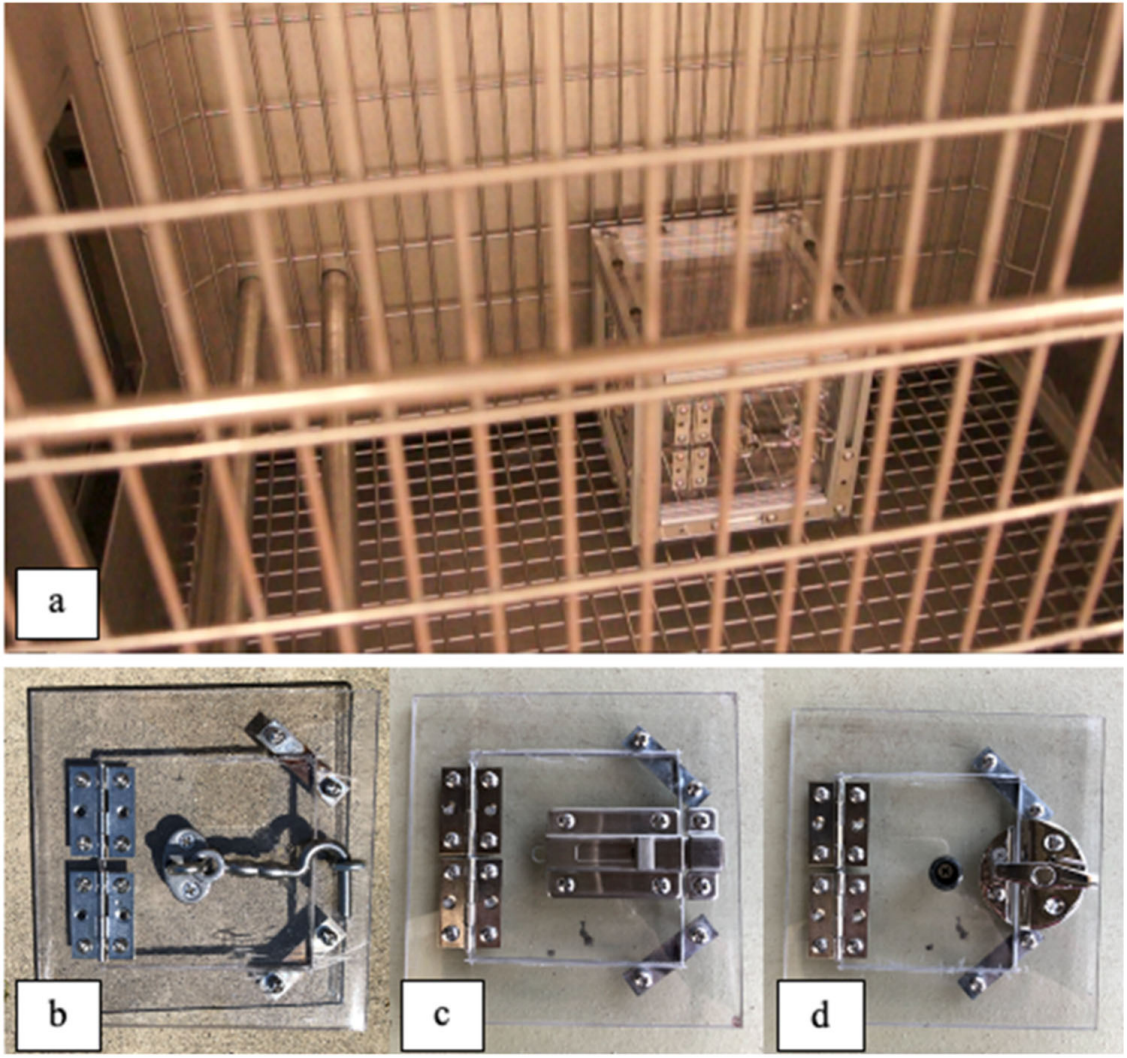
Multi‐access puzzle box placement in the testing cage (a), as well as the three door locking mechanisms used in the experiment (b) hook (c) slide (d) twist.

For this experiment we used a MAB with three solutions on adjacent sides (similar to Daniels et al. 2019). The MAB was fixed to the bottom of the cage during testing and measured 8in × 8in × 8in, with 3in doors on three sides. Each side had a different door latch to open the box and receive the reward (Figure 2). Each solution (hook, slide, twist) required the individual to pull the door towards themselves once the latch was in the open position. Each solution could be locked during testing, rendering that solution obsolete. The side panels of the box were interchangeable within the aluminum frame, allowing for easy randomization of panel orientation across individuals to minimize potential side bias (Guo et al. 2009). A single grape was used as a motivating food reward, which is a common favorite treat amongst our captive rhesus macaque population (Del Rosso, *personal communication*).

### 2.3 Experimental Protocol

Before test trials, we provided all subjects with a habituation trial for acclimation to the device. During habituation trials, the subject was given the MAB with all doors open and a single grape inside. To pass the habituation trial subjects were required to reach inside the box and take the grape reward, after which they moved onto test trials. Neophobia was measured as the latency in seconds from the trial start to when the subject took the grape during habituation.

**Figure 3:**
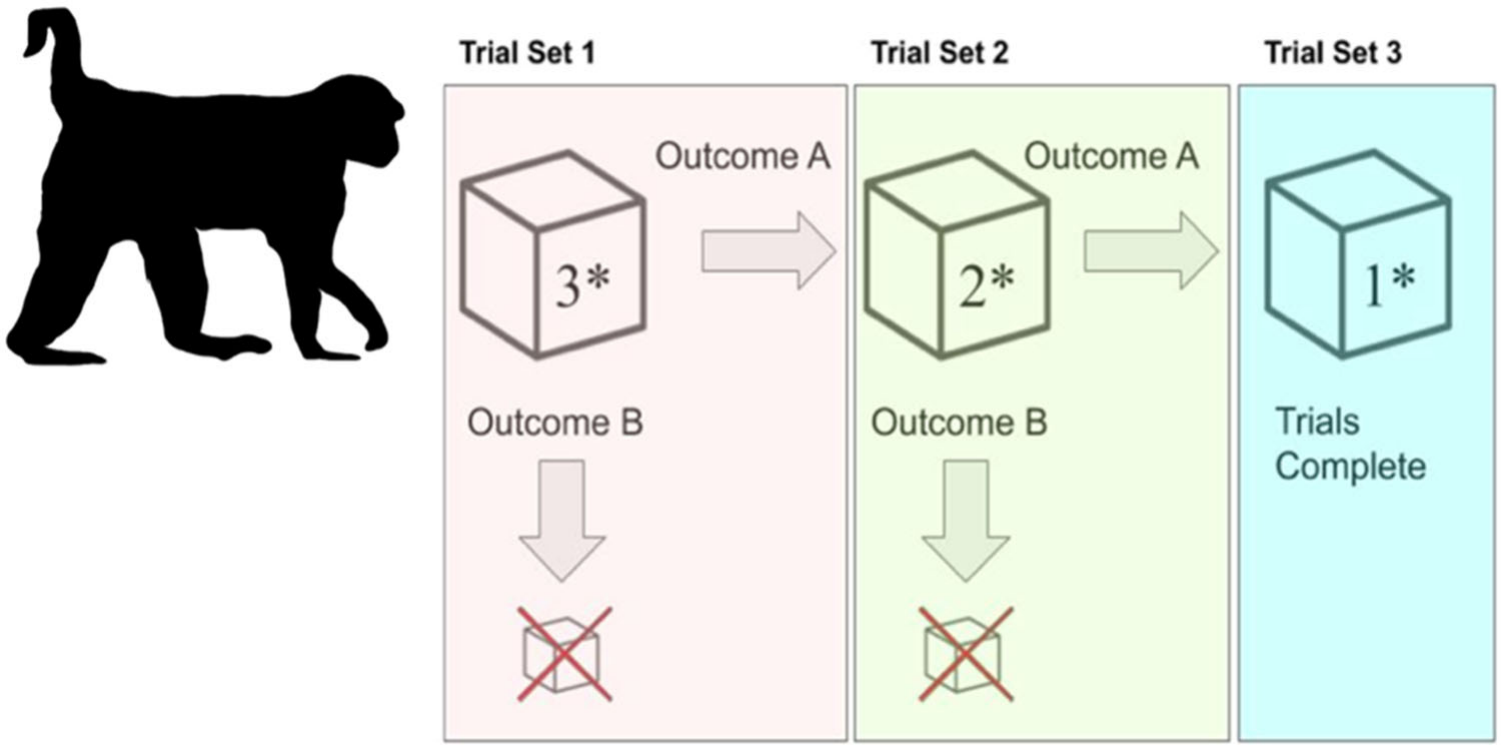
Experimental protocol for the MAB study. Asterisks represent the number of solutions available to solve. Outcome A is when a solution is solved consistently. Outcome B is when a solution isn't solved consistently, resulting in termination of the experiment.

During test trials, individuals were given the MAB with all three doors in the closed but unlocked position (Figure 3). We tested individuals a minimum of three times, and a maximum of six times per day. If an individual solved a door on the MAB, it was rebaited in the same condition (all solutions unlocked) and re‐presented to the individual. Test trials ended either when the subject opened a door, or at a maximum of 10 min. If the individual was in contact with the MAB at the 10‐min mark, an additional 5 min were given to solve the puzzle. If an individual opened the MAB within the first three trials, they continued to receive the MAB until they solved three times using the same solution within a session, or they failed to solve any door in three consecutive trials (Daniels et al. 2019). In only two instances, individuals exceeded the maximum number of six trials due to their continued participation and sequential solving of doors across trials, where one received seven trials, and another received nine. Successful individuals were considered those that solved the MAB a minimum of three times using the same solution. Those individuals would receive the MAB again on a subsequent day, but with the previously preferred solution locked. Thus, individuals needed to open each solution type a minimum of three times each to reach the end of all experimental trials. Although it was rare, if an individual solved multiple solutions within a session, the solution solved most frequently was locked in subsequent trials.

### 2.4 Video Coding and Data Analysis

Videos of MAB solving behavior were coded and analyzed in BORIS from three observers (V. 8.20.4, Friard and Gamba 2016; Cohen's *k* > 0.80 for box directed behaviors, latency to solve, and error counts/durations on Days 2 and 3). Before beginning each experimental trial, unique information was recorded including the date, subject tested, as well as the experimental condition such as habituation, Day 1, Day 2 or Day 3 testing. The trial start time was recorded and the latency for the subject to first touch the MAB (i.e., neophobia). If the subject solved any of the doors of the MAB, the time was recorded when they opened the door as well as when they took the grape from inside the box. The latency to solve a door was calculated as the number of seconds elapsed from the time when the individual first contacted the box to when they opened the door and took the grape. All box‐directed behaviors were extracted and scored as state behaviors with unique motor patterns. Each unique box directed behavior was summed together to calculate exploratory diversity per individual per trial (Table 1), whereas persistence was calculated as the duration in seconds the individual was in contact with the MAB. In addition to box‐directed behaviors, we also recorded other types of behaviors such as aggressive threats directed towards the experimenter (threat frequency) and the presence of stereotypic behaviors as a binary variable. For subjects that consistently solved a door on Day 1 and moved onto Day 2 or Day 3 testing, the number of errors or duration of interacting with a previously solved door was calculated (i.e., inhibitory control).

**Table 1:** Ethogram of MAB‐directed behaviors.

Behavior	Definition
Circle	Focal approaches within arm's length and walks around it while attending to it.
Peer	Focal approaches within arm's length and leans down to look through the sides of the box.
Sniff/Lick	Focal brings their face to the box to contact either with or without the tongue extended.
Touch top	Focal contacts the box by touching the top panel of the box with their hands or feet, including the corners that are adjacent to the top panel.
Touch front	Focal contacts the box by touching the front panel of the box with their hands or feet, including the latching mechanism.
Touch left	Focal contacts the box by touching the left panel of the box with their hands or feet, including the latching mechanism.
Touch right	Focal contacts the box by touching the right panel of the box with their hands or feet, including the latching mechanism.
Bite top	Focal contacts the box with their open mouth on the top panel of the box, including the corners that are adjacent to the top panel.
Bite front	Focal contacts the box with their open mouth on the front panel of the box, including the latching mechanism.
Bite left	Focal contacts the box with their open mouth on the left panel of the box, including the latching mechanism.
Bite right	Focal contacts the box with their open mouth on the right panel of the box, including the latching mechanism.
Climb	Focal lifts their body up perpendicular to the front of the box using their front limbs.
Stand on	Focal climbs on top of the box, including a minimum of three limbs.
Pull/Push	Focal uses their arms to pull or push the box to cause it to rock but not be displaced due to the fasteners.
Cage shake	Focal climbs on top of the box, including a minimum of three limbs, and uses their body weight to cause the entire enclosure to shake.
Raise	Lift the latch vertically to unlock and unhook the metal hook to open.
Slide	Slide the latch horizontally to open.
Twist	Twist the latch 90 degrees clockwise to open.
Pull knob	Pull the door towards the focal to open the door and gain access to the food reward.

### 2.5 Behavioral Predictors

A subset of subjects (*N* = 25) had behavioral data collected when they lived in large social group enclosures several years before testing. These data were collected as a part of the social networks and health project (SNH) from 2013 to 2015 where adult dyadic behaviors were recorded to better understand social interactions and health outcomes in rhesus macaques (McCowan et al. 2016; Balasubramaniam et al. 2018). Dyadic dominance interactions including displacements and aggression were used to calculate ordinal ranks with a network‐based calculation using the Perc package in R (Fujii et al. 2019).

Another subset of test subjects (*N* = 27) had participated in the BBA program when they were young infants. This program separates infants temporarily from their mothers at 3–4 months of age and exposes them to a battery of tests over the course of 25 h (reviewed in Capitanio and Mason 2000). During BBA, infants were assessed on how they acclimated to the testing space by measuring activity levels and emotionality levels at the beginning of the separation and at the end of the testing period (Capitanio and Mason 2000). During the test battery, infants were exposed to two novelty response assessments, a novel object test and a visual paired comparison test. The novel object test measures the proportion of physical interaction with novel objects, while the visual paired comparison measures the proportion of time attending to novel images. At the end of the battery, infants were scored on a Likert scale from 1 to 7 on 16 individual personality metrics. These metrics were then calculated into four scales derived from exploratory and confirmatory factor analyses (calculated and described in Golub et al. 2009): Vigilance, Confidence, Gentleness and Nervousness (Table 2). Scale names are based on the highest, positive loading for each scale.

**Table 2:** Definitions for factor scales of temperament in the BBA program at the CNPRC.

Temperament metric	Definition
Vigilant	Vigilant, NOT depressed, NOT tense, NOT timid
Confident	Gentle, calm, flexible, curious
Gentle	Confident, bold, active, curious, playful
Nervous	Nervous, fearful, timid, NOT calm, NOT confident

### 2.6 Statistical Analysis

We fitted generalized linear models (GLMs) to test differences in innovation and flexibility with the MAB. All statistical assessments were conducted in the program R using the “lme4” and “glmmTMB“ packages (R Core Team 2022, V. 4.3.0). Within each model set (all models testing a single outcome), a Bonferroni correction was applied to reduce the likelihood of false positives by dividing the standard alpha of 0.05 by the number of tests conducted per outcome (See S1 for model details). Predictors included within a model were grouped by research question such as within‐trial behaviors (neophobia, exploratory diversity, persistence, presence of stereotypies, threat frequency), BioBehavioral Assessment measures (nervous score, confident score, vigilant score, gentle score, activity Day 1, activity Day 2, emotionality Day 1, emotionality Day 2) and individual traits (age, health status).For within‐trial behaviors and BBA measures, we first ran a full model with all predictors of interest to assess collinearity using the “performance” package. If predictors were found to be moderately or highly correlated, the variable with the highest variance inflation factor (VIF) was removed from the model and the model was reassessed for collinearity. This process continued until all variables in the model had little to no correlation. Finally, we reduced the number of parameters in the model to determine the best fit model using a backwards stepwise model selection approach using the “stats” package. Additionally, rank was tested in a separate model due to only a subset of animals with rank data (sample sizes by outcome provided in S1). Due to more restricted sample sizes, we did not test the influence of individual traits on the likelihood to solve on Day 2 or Day 3 (*N*_rank_ = 7, N_BBA_ = 9).

Overall, we tested six model sets. For models testing the likelihood to solve a single door consistently on Day 1, Day 2 and Day 3 of testing, we used a binomial distribution. For models testing predictors for overall innovation score and exploratory diversity on the first trial of Day 1, we used a Poisson distribution. For models testing the latency to solve on the first trial of Day 1, we attempted to use a negative binomial distribution due to evidence of overdispersion in the Poisson model. However, even with a negative binomial distribution, all models with the latency to solve outcome continued to show overdispersion, so we removed this outcome from the analysis due to violation of model assumptions. Predictors related to rank were scaled between zero and one to account for variation in group size, where one represented high rank and zero represented low rank. BBA variables were z‐scored within a yearly cohort of animals tested to control for interannual variation.

### 2.7 Ethics and Approval Declaration

The protocols used in this study were approved by the Institutional Animal Care and Use Committee (IACUC) of the University of California, Davis (protocol #22691). The research was performed strictly in accordance with the guidelines drafted in this protocol and complied with the legal requirements of National Primate Research Facilities and NIH regulations. This protocol, along with the guidelines and regulations, was designed in consultation with the animal care staff and behavioral management unit of the CNPRC in Davis, California, USA.

## 3 Results

### 3.1 Overall Multi‐Access Puzzle Box Performance

Out of the 50 females tested, forty‐seven of them passed habituation by taking the grape during the initial open condition (Table 3). Of those that moved onto the testing phase, twenty‐one females solved at least one door. Seventeen females solved a door consistently and moved onto Day 2 testing. On Day 2, 10 individuals solved a second door with 9 of those solving a second door consistently allowing them to move onto Day 3 testing. On Day 3, seven animals solved a third door, with six of them solving a third door consistently, completing the testing regime. Although most individuals had short time lags between testing days (2 days max), there was one female that had a longer lag in testing (> 3 days) between Day 2 and Day 3. Although this female solved consistently on both Day 1 and Day 2, they did not solve on Day 3. It is hard to discern whether this lag in testing contributed to the lack of solving on Day 3 for this individual as it is possible that they may have lost the association between door solves across the interim period between Day 2 and Day 3 testing sessions.

**Table 3:** MAB testing conditions including total sample size (*N*), number of solvers and consistent solvers by solution type, average solve time and standard deviation in seconds.

Condition	*N*	Solved a door	Solved repeatedly	Hook	Slide	Twist	(Majority) %	Avg. solve time (sec)	SD (sec)
Habituation	50	N/A	N/A	N/A	N/A	N/A	N/A	N/A	N/A
Day 1	47	21	17	14	3	0	(Hook) 82%	134.72	150.01
Day 2	17	10	9	1	5	3	(Slide) 55%	151.5	111.95
Day 3	9	7	6	1	2	3	(Twist) 50%	273.5	254.37

Individuals varied in which doors they solved on different testing days. On Day 1 most individuals solved the hook door, whereas a few solved the slide door (Table 3). However, on Day 2 when most individuals no longer had access to the hook door because it was locked, the second door solved was more equally split between slide and twist (55%–50%). Overall, twist was solved much less often (6/32 solved trials) compared to other door mechanisms such as slide (10/32) and hook (16/32). Due to the uneven distribution of solution types, we looked at the average solve time per door to get an idea of differences in potential difficulty across solution types. We found that on Day 1 of testing, subjects were on average quicker to solve the hook solution, followed by the slide and twist solutions, with a significant amount of variation in solve time for all three solutions (Table 3).

### 3.2 Within‐Trial Behaviors and MAB Performance

**Figure 4:**
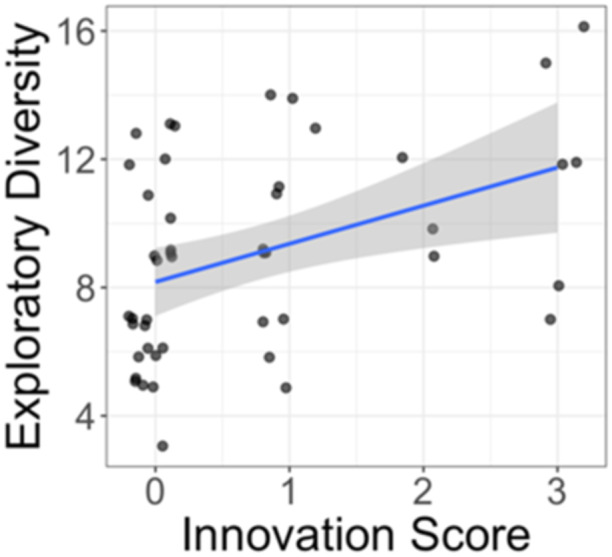
Exploratory diversity scores by overall innovation score across repeated presentations of the MAB.

For within‐trial behaviors, we found that those with higher exploratory diversity also had higher innovation scores (Figure 4; *β* = 0.25, *p* = 0.0001) and greater likelihood to solve on Day 1 (*β* = 0.53, *p* = 0.001). We calculated interindividual variation in exploratory diversity to better understand its potential role in MAB solving behavior, which showed significant variation on the first trial of Day 1, (range = 3–16, mean = 9.08, SD = 3.13). For persistence, we found that those that worked longer on the MAB had higher exploratory diversity scores (*β* = 0.001, *p* = 0.0003), albeit with a small effect size. However, neophobia did not show any relationships with the outcomes tested and was dropped from the best fit models (Supporting Information S1: S1).

### 3.3 Individual Traits and MAB Performance

**Figure 5:**
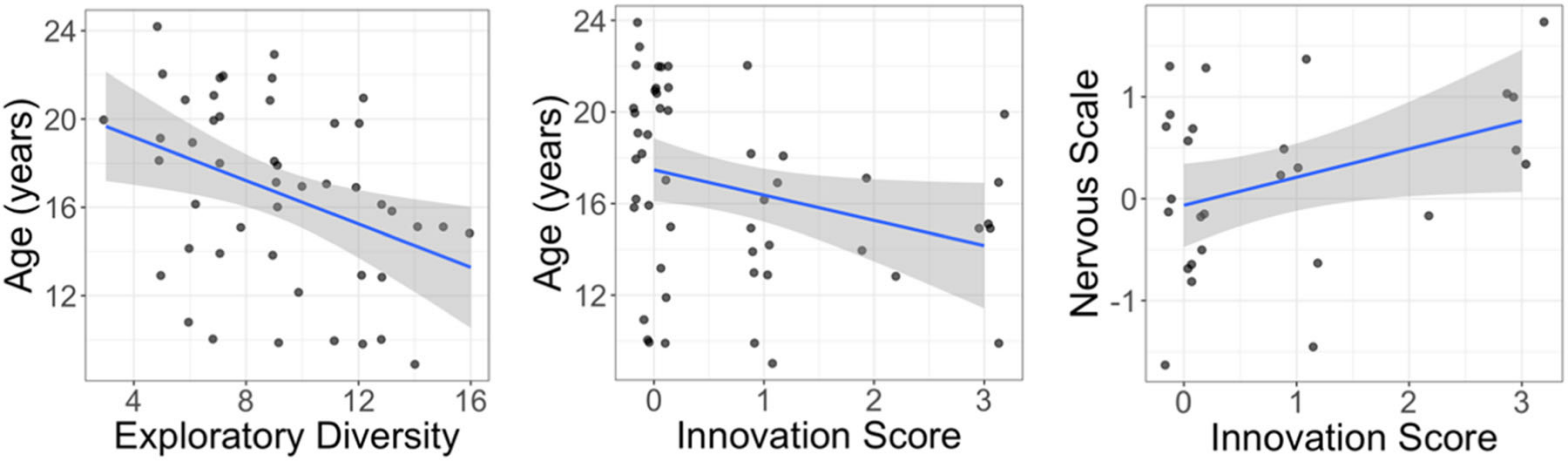
Influence of age on exploratory diversity and overall innovation score as well as the influence of the personality metric nervous on overall innovation score.

We tested the influence of individual traits on overall innovation score, exploratory diversity score and the likelihood to solve on the first day of testing. For age, we found that younger adults were more likely to have a higher innovation score (Figure 5; *β* = −0.11, *p* = 0.011) and had higher exploratory diversity scores than older adults (*β* = −0.03, *p* = 0.004). However, we did not find any relationship between age and likelihood to solve on Day 1 (*β* = −0.17, *p* = 0.037). We also did not find any relationships between health status and innovation score, exploratory diversity or likelihood to solve on day 1 (Innovation score *β* = −0.88, *p* = 0.023; Exploratory diversity *β* = −0.16, *p* = 0.091; Solve Day 1 *β* = −1.16, *p* = 0.100). We also did not find any relationships between rank and innovation score, likelihood to solve on Day 1 or exploratory diversity (Innovation score *β* = 0.23, *p* = 0.854; Solve Day 1 *β* = −1.18, *p* = 0.634; Exploratory diversity *β* = −0.16, *p* = 0.134). For BBA metrics, we found that females that scored high on nervous temperament ratings had higher innovation scores (Figure 5; *β* = 0.95, *p* = 0.006). However, we did not find any relationships between other BBA metrics and exploratory diversity or the likelihood to solve on day 1 with most variables being dropped from the best fit models (S1).

### 3.4 Inhibitory Control in MAB Performance

For individuals that solved on Day 1, they moved onto Day 2 for additional testing (*N* = 17). To test inhibitory responses to the MAB, we calculated the number of errors the individuals made on locked solutions as well as the duration of time spent on locked solutions. However, we did not find any relationships between either the frequency or duration of errors and the likelihood to solve on either Day 2 (frequency β = −0.02, *p* = 0.42; duration *β* = −0.005, *p* = 0.315) or Day 3 (frequency *β* = −0.01, *p* = 0.877; duration *β* = 0.001, *p* = 0.939).

## 4 Discussion

Using a MAB design, we investigated how individual traits and within‐trial behaviors predicted repeated innovation in rhesus macaques (*Macaca mulatta*). Overall, exploratory diversity was a major predictor of innovation and repeated innovation across three problems on the MAB. Individual traits such as age and personality metrics were also observed to predict repeated innovation. Younger adults were more successful at repeated innovation and had higher exploratory diversity scores than older adults. Additionally, adults that scored high on nervousness in a personality assessment during infancy were better at repeated innovation as adults. However, we did not find any effects of inhibitory control, as measured by the number of errors or duration of time spent on previously locked solutions, on the likelihood to solve on Day 2 or Day 3.

### 4.1 Overall MAB Performance

In captivity, rhesus macaques have been observed to work voluntarily for regularly provided foods such as monkey chow (Reinhardt 1994). However, even though most individuals in this study passed the habituation criterion of taking the grape during the open condition, we observed a relatively low rate of solving on Day 1 (17/50 or 34% of subjects). Future studies on MAB performance in macaques may consider testing subjects that are more often foraging in a complex environment or need to make more frequent foraging decisions. It is possible that the low solving rate in our sample was due to the indoor pair‐housing conditions of the animals, which is relatively simple compared to a wild environment. Future studies may also benefit greatly from manipulating foraging complexity of test subjects before MAB testing to better understand the relationship between environmental complexity and motivation to work on novel foraging puzzles or exploratory tendencies. For example, studies that have provided environmental enrichment to rhesus macaques in small social groups have found it to increase overall exploratory behaviors (Byrne and Suomi 1991). Additionally, studies may also benefit from conducting a set of food preferences before beginning with MAB testing. Our study works under the assumption that grapes are a widely accepted and loved treat for macaques because they are often only used for special circumstances. However, we do know that individuals can hold unique food preferences (Martin et al. 2018), so controlling for this potential condition would help to confirm the motivational state of the subject at the time of testing. In combination with other knowledge of rhesus behavior towards novel food puzzles under different housing and social conditions, our current study provides evidence that captive adult females are motivated to participate in physical cognitive tasks and that they show variation in their ability to repeatedly innovate on the MAB.

Out of all individuals tested, less than half were able to solve a single door consistently on Day 1, with the favored solution being the hook mechanism. This may be due to the hook solution being easier to solve than slide or twist, as evidenced by the shorter average solve time across individuals (Table 3). However, it is notable that there was significant variation observed in solve time across all three solutions, which could be attributable to differences in sample size or individual variation in solving behaviors. Studies focused on understanding the role of individual variation on innovation success focus on comparing tasks that are expected to have similar levels of difficulty across subjects. However, practically designing these studies is complex as it can be challenging to align experimenter expectations of task difficulty with actual difficulty for subjects until measures have been validated. Perhaps a two‐stage experimental protocol may prove useful in these situations, where during the first stage one set of animals are tested on a variety of solving mechanisms to discern which are of similar difficulty as measured by latency to solve. In the second stage, a separate group of animals could be tested on a MAB with only validated solutions from stage one to tease apart the role of individual differences from task difficulty.

During test trials, behaviors thought to distract the subject from the MAB were measured to assess their influence on repeated innovation. Behaviors such as frequency of threats towards the experimenter may reflect aspects of reactivity of those individuals, which could prove to be inhibiting for innovation performance. Interestingly, despite the fact that greater emotional reactivity has been shown to negatively influence participation on cognitive tests in other primate species, it does not appear to influence overall success on the task (common marmosets: Schubiger et al. 2015). Although we did not test the influence of emotional reactivity on likelihood of participation in the task, we did find that emotional reactivity in macaques, as measured by threats directed towards the experimenter, did not influence performance on the MAB. Additionally, the presence or frequency of stereotypical behaviors can indicate aspects of mental well‐being and coping skills, which if performed with great frequency or duration, can take time away from focusing on solving the MAB. However, the relationship between problem solving and stereotypical behaviors is complex, and often not easily predicted. For example, a study on three different foraging puzzles or enrichment devices were provided to captive macaques to reduce stereotypical behavior. However, the rates of stereotypies were variable depending on the puzzle feeder deployed, suggesting that traits of the puzzle feeders themselves may induce different coping strategies or mechanisms (Gottlieb et al. 2011). Furthermore, the fact that individuals that displayed stereotypies in our study were not inhibited to solve the MAB on Day 1 or across days suggests that stereotypical behavior may function effectively as a manifestation of coping skills to overcome stressful scenarios (such as being presented with a novel object) to achieve success on an innovation task.

### 4.2 Within‐Trial Behaviors and MAB Performance

In this study, exploratory diversity was found to be the most crucial predictor of repeated innovation, as well as the likelihood to solve on the first day of presenting the MAB. This suggests that exploratory diversity is particularly important when individuals are confronting novel problems. These results have been commonly reported in the literature assessing repeated innovation using a MAB across species. For example, across mammals, researchers have found that higher exploratory diversity scores significantly influenced repeated innovation in a MAB task (yellow‐bellied marmots: Williams et al. 2021; spotted hyenas: Johnson‐Ulrich et al. 2018; bat‐eared foxes: Petelle et al. 2023; raccoons: Daniels et al. 2019). However, these results are not universal across all species considering that a study in Asian elephants found persistence to be more indicative of repeated innovation rather than exploratory diversity (Jacobson et al. 2021). Thus, it is interesting that exploratory diversity in this study was shown to be more indicative of repeated innovation on the MAB compared to persistence, considering that persistence has been shown to be important for a variety of other species as well (bat‐eared foxes: Petelle et al. 2023; Johnson‐Ulrich et al. 2018; Daniels et al. 2019). One potential explanation for this is that many of these studies did not explore the predictors of exploratory diversity itself, but rather only explored how exploratory diversity compared to other metrics such as persistence on innovation success. However, when we tested what predicted exploratory diversity in macaques, we found that persistence was the most significant component. This suggests that for macaques, the amount of time that an individual works on a problem, the more likely they are to express a wider diversity of behaviors towards the MAB. However, we recognize that the relationship between persistence and exploratory diversity may not always be positive, since animals who persistently use the same action patterns to interact with a task may have low diversity while still maintaining high persistence. Due to differences in evolutionary pressures, we may expect different species to show different relationships between persistence and exploratory diversity when interacting with novel objects. We propose that macaques may be less likely to repeat previously used actions when persisting on a task due to higher rates of learning and intelligence observed in these species. These investigations highlight the important roles that persistence and exploratory diversity play in repeated innovation, where persistence for working on a problem may allow individuals to express a greater number of behaviors towards the object, thus increasing their likelihood to solve repeated problems. Additionally, although the literature on whether neophobia influences repeated innovation is mixed, we did not find any influence of neophobia on repeated innovation in macaques.

### 4.3 Individual Traits and MAB Performance

This study only tested for repeated innovation in adult female macaques rather than across both sexes due to limitations in accessibility to males for testing at our facility. Considering that sex differences have been shown to have significant influences on cognitive performance both in human children and in monkeys (Overman et al. 1996), this may have important implications for interpreting our results more broadly. Indeed, a review on reports of innovation in nonhuman primates revealed that innovation is more often reported for males and adults, rather than females and nonadults, than would be expected by chance given the relative proportions of those groups (Reader and Laland 2001). In chimpanzees, which were the only group large enough to investigate alone, they found a similar pattern with males showing greater rates of innovation than females (Reader and Laland 2001). Thus, given that our study includes only females, we might expect this to represent a lower limit of innovation rates for macaques given overall trends in primates for higher innovation rates in males. Other studies on rhesus macaques have found sex differences in the ability to exert inhibitory control in a battery of touch‐screen tasks with females outperforming males on some measures (Loyant et al. 2021). Although it is unclear how these digital inhibitory tasks map onto physical inhibitory tasks such as responses to locked solutions on the MAB, one might expect that if the MAB is truly measuring inhibition, then it would positively correlate with digital measures of inhibition. Thus, we might expect that if we included males in our study that females would have outperformed them at later stages of the experiment testing repeated innovation and inhibitory control on locked solutions. Future studies focused on repeated innovation and inhibitory control in macaques should test these ideas by incorporating balanced samples of both sexes.

Regarding the predictability of individual traits on MAB performance, age was a strong predictor of repeated innovation and exploratory diversity. Younger adults were more likely to repeatedly innovate and had higher exploratory diversity scores. Although previous research on innovation in macaques has shown that adults are more likely to solve than juveniles (Mangalam and Singh 2013), no studies to our knowledge have investigated age as a continuous variable across the adult age class. Our result that younger adult females appear to be better at repeated innovation suggests that flexibility to solve multiple solutions may peak in early adulthood. This suggests that innovation (or repeated innovation) and age in macaques may assume a U‐shaped relationship, where young individuals are not yet skilled or dexterous enough to solve, whereas older adults are either unable to solve or are not motivated to try. Future studies should aim to disentangle the relationship between innovation, exploration and inhibition across different life stages in development to further test these ideas.

Behavioral ecology theories posit that different environmental pressures may select for the expression of behaviors such as dominance among conspecifics. Under normal social conditions for macaques, high ranked individuals may not experience strong pressures to work on foraging problems due to priority of access to preferred resources and the need to oversee and maintain social group dynamics. For example, high ranked individuals might spend a considerable amount of time keeping track of the social interactions of others instead of spending time innovating on foraging problems. Animals living under captive conditions with small group sizes or pair‐housing such as the subjects in this study may allow for individuals to express their natural exploratory tendencies upon release from time constraints, foraging costs, and social pressures in the wild. However, despite the theoretical importance of rank for innovation success, we did not find any relationship between rank and repeated innovation on the MAB. This may be due to the fact that there was a long period of time between when the rank data were collected and when individuals were tested with the MAB (range: 7–9 years). Although rank orders in primates have been shown to be relatively stable across years (Shively and Kaplan 1991; Mori et al. 1989), the change from outdoor social housing to indoor pair‐housing may extinguish the importance of rank variables for individuals, thus rendering it obsolete. Furthermore, replicating these studies with rank data that is collected closer to the time of experimental testing as well as more directly comparable between captive and wild conditions would be an interesting addition to the literature.

Aspects of personality have been proposed to be shaped by ecology and evolution to influence different cognitive styles, the likelihood of success in cognitive tasks, as well as success in cognitive tasks across the lifespan (Capitanio and Mason 2019; Carere and Locurto 2011). However, studies assessing the influence of personality on repeated innovation success are largely missing from the literature. Additionally, there are a variety of ways to measure personality including open field tests, responses to novelty and experimenter ratings. Due to this it can be difficult to predict how personality measures may influence innovation. In a recent meta‐analysis of predictors of innovation across animals, it was found that species that are more neophilic and more exploratory in response to open field tests and novel objects were found to innovate more often (Amici et al. 2019). We predicted that individuals who scored high on confidence or gentle personality metrics (similar to boldness measures) were more likely to innovate or repeatedly innovate on the MAB. These predictions were informed in part by previous MAB studies that had investigated personality metrics finding that nervous or cautious individuals were less successful in solving a MAB (O'Connor and Vonk 2022). However, we found that for rhesus macaques the personality factor of nervousness was most indicative of repeated innovation, where those that scored high on nervousness during infancy were more likely to repeatedly innovate on the MAB as an adult. This contradictory finding may be because more nervous animals often express other traits such as high levels of activity, which has been shown to influence repeated innovation in other species (Johnson‐Ulrich et al. 2018). This is perhaps indicated by our results that other variables were nearly‐significant when tested in the model along with personality metrics, where level of activity on Day 1 of the infant relocation positively predicted repeated innovation (Supporting Information S1: S1). Although nervous temperament outperformed activity levels in the model, this may suggest that high rates of activity in response to a relocation as infants could manifest as higher rates of overall activity as adults, which may facilitate innovation or repeated innovation. Another consideration is that nervousness may also manifest in different ways, where some species express high rates of activity or flight behavior, while others may reduce activity or freeze in response to stressors. Additionally, individuals who score high on nervousness may also share traits such as timidness that align with the slow but methodical cognitive phenotype compared to fast and impulsive learners (Madden et al. 2018; Guillette et al. 2017). Although fast learners may be associated with exploration and boldness, this could also lead to impulsive and inaccurate responses, whereas slow learners may be more methodical and accurate (Šlipogor et al. 2022). In fact, fast versus slow learners can even influence flexibility when contingencies change, where in Indian mynas faster innovators were found to be slower to change behaviors when cues about food sources changed (Griffin et al. 2013). These varied findings across species may reflect the complex influence of personality on cognition. A recent meta‐analysis found highly variable directions for personality metrics across studies and their influence on cognitive outcomes across species (Dougherty and Guillette 2018). We hope that these results encourage future researchers to explore personality metrics when assessing problem‐solving, innovation or repeated innovation to better disentangle these effects within primates and across the animal kingdom.

### 4.4 Inhibitory Control in MAB Performance

It is curious that our measures of flexibility did not have any predictive power for repeated innovation on Days 2 or 3. It is hard to discern whether these results reflect that flexibility truly does not play an important role in repeated innovation for macaques or whether there are other ways that we could have measured flexibility that may better represent these processes. Some studies on innovation and flexibility have looked at the intersection between neophobia, problem‐solving ability, and flexibility using separate unique tests (Logan et al. 2022; Logan et al. 2023) and although some have found relationships between these independent assays, others have found mixed results. For example, another classic test of flexibility is the detour task, where subjects must inhibit their immediate impulse to grab food directly out of a clear tube, and instead reach around to the side of the clear tube to grab the food reward (Amici et al. 2008). Adding additional measures of flexibility such as the detour task may better help us discern the validity of independent flexibility assessments as well as whether there is consistency in performance across tasks aimed at measuring flexibility. Furthermore, additional assessments that may not measure flexibility directly but may contribute to the expression of flexibility may also prove useful in understanding the role of flexibility in solving novel foraging problems. For example, independent novel object testing both with and without food rewards would be a worthy contribution aimed at teasing apart general exploratory tendencies with novel objects rather than exploratory tendencies with novel food objects, and the role of these traits with behavioral flexibility expression.

## Author Contributions

**Josephine Hubbard:** conceptualization (equal), data curation (lead), formal analysis (lead), investigation (lead), methodology (lead), project administration (equal), validation (lead), visualization (lead), writing – original draft (equal). **Brenda McCowan:** conceptualization (equal), funding acquisition (lead), project administration (equal), resources (lead), writing – original draft (equal).

## Supporting information

Supporting information.

## Data Availability

The data that support the findings of this study are available from the corresponding author upon request.
