# Supplementary material for: Innovation, Inhibition and Flexibility in Rhesus Macaques (Macaca mulatta)
Source: Am J Primatol. 2025 Mar 30;87(4):e70027. doi: 10.1002/ajp.70027 (PMC11955746; doi:10.1002/ajp.70027)
Supplement: Supplementary file 1 — Supporting information. [file AJP-87-e70027-s001.pdf]

AJP_Hubbard&McCowan_Innovation, inhibition and flexibility in rhesus macaques (*Macaca mulatta)*

Outcome: Innovation Score (0-3)		N=50; Nrank= 25; Nbba=27			a=0.05	a=0.0125
**Model formula**	**AICc**	**Predictors**	**Estimat**	**SE**	**p**	**adjusted p**
Innovation_Score ~ 1 , data = MAB_Data, family = "poisson"	123.39	Intercept	-0.32	0.16	0.048	0.048
Innovation_Score ~ Rank, data = MAB_Data, family = "poisson"	59.77	Intercept	-0.63	0.71	0.379	0.379
		Rank	0.23	1.30	0.854	0.854
Innovation_Score ~ ExploratoryDiversityDay1Trial1 + PersistenceI	107.81	Intercept	-2.04	0.62	0.001	0.001
		Exploratory Diversity	0.25	0.06	**0.0001**	**0.0001**
		Persistence	-0.01	0.002	**0.028**	0.028
Innovation_Score ~ RoundedAge + HealthStatus, data = MAB_Dat	116.43	Intercept	1.80	0.73	0.013	0.013
		Age	-0.11	0.04	**0.011**	**0.011**
		HealthStatusSPF	-0.88	0.39	**0.023**	0.023
Innovation_Score ~ BBA_Nervous + Avg_PropNovelLook + BBA_D	62.61	Intercept	0.35	0.84	0.673	0.673
		Nervousness	0.95	0.34	**0.006**	**0.006**
		Proportion Novel Loo	-2.28	1.36	0.094	0.094
		Day1Activity	0.78	0.32	**0.015**	0.015
		Day2Emotionality	-0.48	0.30	0.106	0.106

Outcome: SolveDay1 (Y/N)		N=47; Nrank=23, Nbba=26			a=0.05	a=0.0125
**Model formula**	**AICc**	**Predictors**	**Estimat**	**SE**	**p**	**adjusted p**
SolveDay1 ~ 1 , data = MAB_Data_HabituationPassOnly, family =	63.51	Intercept	-0.56	0.30	0.061	0.061
SolveDay1 ~ Rank, data = MAB_Data_HabituationPassOnly, famil	**32.63**	Intercept	-0.21	1.34	0.872	0.872
		Rank	-1.18	2.49	0.634	0.634
SolveDay1 ~ ExploratoryDiversityDay1Trial1 + PersistenceDay1Tri	**53.07**	Intercept	-3.80	1.27	0.002	0.002
		Exploratory Diversity	0.53	0.16	**0.001**	**0.001**
		Persistence	-0.01	0.01	**0.014**	0.014
SolveDay1 ~ RoundedAge + HealthStatus, data = MAB_Data_Habi	**61.37**	Intercept	2.78	1.48	0.061	0.061
		Age	-0.17	0.08	**0.037**	0.037
		Health Status	-1.16	0.70	0.1009	0.101
SolveDay1 ~ BBA_Gentle + BBA_Vigilant + Avg_PropNovelObj + A	**32.30**	Intercept	5.70	3.85	0.138	0.138
		Nervous	1.65	0.80	**0.039**	0.039

		Proportion Novel Loo	-12.65	6.90	0.069	0.069
		Day1Emotionality	-1.78	1.06	0.094	0.094

Outcome: Exploratory Diversity (0-16) N=47; Nrank=23, Nbba=26 a=0.05 a=0.0125

Model formula	AICc	Predictors	Estimate	SE	p	adjusted p
ExploratoryDiversity ~ 1 , data = MAB_Data_HabituationPassOnly	240.54	Intercept	2.20	0.04	<0.001	<0.001
ExploratoryDiversity ~ Rank, data = MAB_Data_HabituationPassC	**116.26**	Intercept	1.86	0.21	<0.001	<0.001
		Rank	0.57	0.38	0.134	0.134
ExploratoryDiversity ~ Neophobia_LatGrapeHab + PersistenceDay	**227.60**	Intercept	2.02	0.10	<0.001	<0.001
		Neophobia	-0.001	0.004	0.079	0.079
		Persistence	0.001	0.001	**0.0003**	**0.0003**
ExploratoryDiversity ~ RoundedAge + HealthStatus, data = MAB_I	**235.13**	Intercept	2.84	0.21	2.00E-16	<0.001
		Age	-0.03	0.01	**0.004**	**0.004**
		Health Status	-0.16	0.10	0.09	0.091
ExploratoryDiversity ~ BBA_Gentle, data = MAB_Data_Habituatio	**138.46**	Intercept	2.19	0.07	2.00E-16	<0.001
		Gentle	-0.12	0.06	0.061	0.061

Outcome: SolveDay2 (Y/N) N=17 a=0.05 a=0.025

Model formula	AICc	Predictors	Estimate	SE	p	adjusted p
SolveDay2 ~ 1 , data = MAB_Data_HabituationPassOnly, family =	**11.92**	Intercept	3.91	1.01	0.0001	0.0001
SolveDay2 ~ Day2ErrorCount , data = MAB_Data_HabituationPas:	27.68	Intercept	0.55	0.71	0.437	0.437
		Day2ErrorCount	-0.02	0.02	0.402	0.402
SolveDay2 ~ Day2ErrorDuration , data = MAB_Data_HabituationF	27.27	Intercept	0.66	0.73	0.363	0.363
		Day2ErrorDuration	-0.01	0.01	0.315	0.315

Outcome: SolveDay3 (Y/N) N=9 a=0.05 a=0.025

Model formula	AICc	Predictors	Estimate	SE	p	adjusted p
SolveDay3 ~ 1 , data = MAB_Data_HabituationPassOnly, family =	**12.10**	Intercept	1.25	0.80	0.118	0.118
SolveDay3 ~ Day3ErrorCount , data = MAB_Data_HabituationPas:	15.51	Intercept	1.48	1.71	0.399	0.388
		Day3ErrorCount	-0.01	0.09	0.877	0.877
SolveDay3 ~ Day3ErrorDuration , data = MAB_Data_HabituationF	15.52	Intercept	1.14	1.62	0.482	0.482

	Day3ErrorDuration	0.00	0.02	0.939	0.939
